# Relationship between birth weight to placental weight ratio and major congenital anomalies in Japan

**DOI:** 10.1371/journal.pone.0206002

**Published:** 2018-10-22

**Authors:** Ryuichi Takemoto, Ai Anami, Hiroshi Koga

**Affiliations:** 1 Department of Pediatrics, National Hospital Organization Beppu Medical Center, Beppu, Oita, Japan; 2 Department of Obstetrics and Gynecology, National Hospital Organization Beppu Medical Center, Beppu, Oita, Japan; BC Children's Hospital, CANADA

## Abstract

Recent studies have indicated that birth weight to placental weight (BW/PW) ratio is related to perinatal outcomes, but the effect of congenital abnormalities on BW/PW ratio remains unclear. We performed this study to elucidate correlations between BW/PW ratio and congenital abnormalities. Subjects were 735 singleton infants born at 34–41 weeks of gestation admitted to our center between 2010 and 2016. Of these, 109 infants (15%) showed major congenital anomalies. Major congenital anomalies and subgroups were diagnosed according to European Surveillance of Congenital Anomalies criteria. The primary outcome was the association between BW/PW ratio and major congenital anomaly, and secondary outcomes were the distribution pattern of BW/PW ratio with major anomalies and by major anomaly subgroups in each categorization (<10th percentile, 10–90th percentile, or >90th percentile) of BW/PW ratio. BW/PW ratio was not associated (*P* = 0.20) with presence (adjusted mean BWPW ratio = 5.02, 95% confidence interval [CI] 4.87–5.18) or absence (adjusted mean BW/PW ratio = 4.91, 95%CI 4.85–4.97) of major anomalies, after adjusting for gestational age and sex. Proportions of infants with major anomalies according to BW/PW ratio categories were as follows: 12% in <10th percentile, 15% in 10–90th percentile, and 25% in >90th percentile of BW/PW ratio. Among major anomalies of the nervous system, congenital heart defects, and orofacial clefts, BW/PW ratio showed equally distributed trend across the three BW/PW ratio categories, but showed unequally distributed trend for anomalies of the digestive system, other anomalies/syndromes, or chromosomal abnormalities. BW/PW ratio was not associated with major congenital anomaly, and was distributed diffusely according to major anomaly subgroups. Major anomalies may tend to aggregate in the 90th percentile of the BW/PW ratio.

## Introduction

Since the 1990s, researchers have been interested in placental weight (PW), and have reported associations between PW and perinatal outcomes [1,2] and the development of diseases in adult life [3]. Eutherian (placental) mammals show a close relationship between PW and fetal growth, and the full-term birth weight (BW) of humans, pigs and goats is approximately five times the PW [4–6]. Human PWs and full-term BWs vary by more than 15% between different races or countries [4,7,8]. However, the full-term BW-to-PW (BW/PW) ratio has been shown to only differ by less than 5% between ethnicities or country of birth [4,7,9]. This suggests that the BW/PW ratio may offer a valuable international perinatal index. A relatively high BW/PW ratio indicates insufficient placental oxygen supply to the fetus. In contrast, a low BW/PW ratio suggests a suboptimal fetal condition. Previous studies have demonstrated associations of BW/PW ratio with perinatal outcomes [10], risk of cerebral palsy [11] and disease outcomes in subsequent adulthood [12]. Although congenital anomalies can affect fetal growth [13], the association between congenital anomalies and PW has yet to be elucidated [14,15]. We hypothesized that fetal congenital anomaly may lead to a low BW/PW ratio because of fetal growth restriction, or to a high BW/PW ratio because of inappropriate fetal overgrowth. We investigated whether associations existed between BW/PW ratio and major congenital anomalies as well as the major anomaly subgroups.

## Materials and methods

### Study design and participants

This cross-sectional study involved singleton infants born at 34–41 weeks of gestation and admitted to the neonatal intensive care unit (NICU) at Beppu Medical Center in Japan, between 1 April 2010 and 31 March 2017. Infants without appropriate measurement of PW were excluded from the study. Written informed consent was obtained from the parents for experimentation with human subjects and the ethics committee at Beppu Medical Center approved this study protocol and consent procedure.

Eligible infants were classified into those diagnosed with major anomalies and those without any major congenital anomaly. Major congenital anomalies were diagnosed and sub-classified according to European Surveillance of Congenital Anomalies (EUROCAT, version 2014) [16]. The diagnostic and classification process is shown in S1 Fig. Minor congenital anomalies were not assessed in this study [17].

The primary outcome measure was the BW/PW ratio, which was categorized into three groups: <10th percentile, 10–90th percentile, and >90th percentile [9]. Secondary outcome measures were the distribution and subgroups of major anomalies according to the three categories of the BW/PW ratio.

PW was measured on a digital scale within 1 hour after delivery along with the membrane and umbilical cord, after removing blood clots [9]. Perinatal clinical information was identified, and neonatal screening (including physical examination, X-ray and ultrasonography) was performed to detect congenital anomalies. Further diagnostic workups, such as computed tomography, magnetic resonance imaging, chromosomal testing, or other genetic testing, were performed by neonatologists as required.

### Statistical analysis

The Levene’s test was used to evaluate the distribution of continuous variables. Student’s t-test was used to assess differences between two groups, as the data were normally distributed. For categorical variables, either a chi-square test or Fisher’s exact test was used, as appropriate. Gestational age and sex were defined as covariates of the BW/PW ratio [4,7,9]. Data were analyzed by analysis of covariance (ANCOVA) after adjusting for these covariates. All statistical analyses were conducted using SPSS Statistics version 20 (IBM, Armonk, NY).

## Results

**Fig 1:**
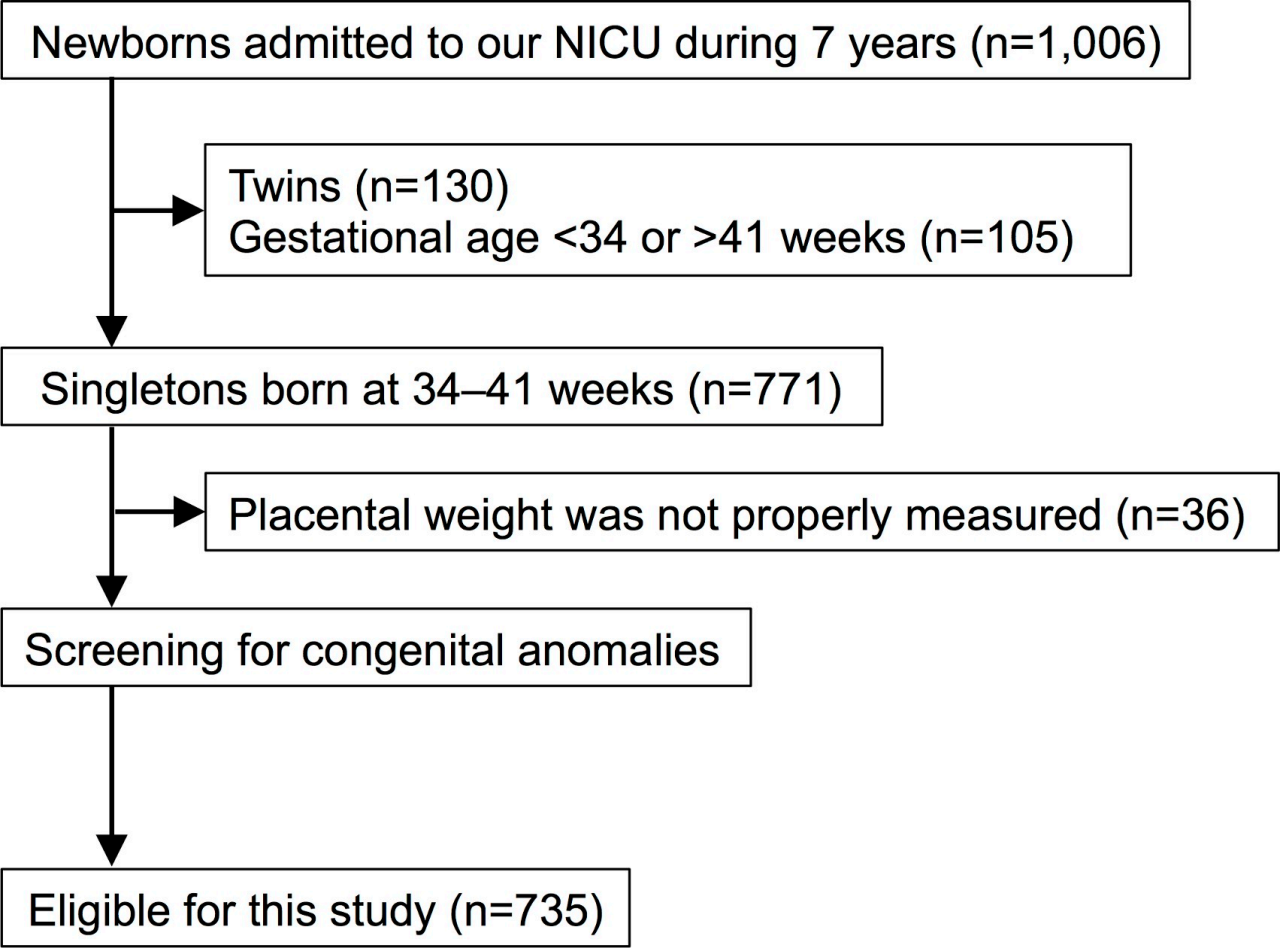
Flowchart for selection of participants.

Infants and placentas from 735 singleton deliveries were enrolled in this study from April 2010 to March 2017, as detailed in Fig 1. The primary reasons for the 735 NICU admissions were as follows: low birth weight infant, n = 245; hyperbilirubinemia, n = 194; congenital anomaly, n = 94; respiratory distress, n = 68; neonatal asphyxia, n = 37; hypoglycemia, n = 35; vomiting, n = 21; infection, n = 14; neurological disorder, n = 7; and other reasons, n = 20. Major anomalies were identified in 109 (15%) of the 735 infants.

The basic characteristics of participants are shown in Table 1. A predominance of females, longer gestational period, higher BW, and an increased BW/PW ratio were all observed in infants born with major anomalies. Diagnoses of major anomalies and associated anomalies according to major anomaly subgroups are listed in Table 2. Of the 109 infants diagnosed with a major anomaly, congenital heart defects were identified in 37 infants (34%), chromosomal abnormalities in 18 infants (17%), other anomalies/syndromes in 17 infants (16%), orofacial clefts in 12 infants (11%) and digestive system abnormalities in 10 infants (9.2%). These five subgroup categories accounted for 82 (86%) of all 109 infants with major anomalies. The most common single major anomaly was ventricular septal defect (VSD), in 25 infants (23%). Down syndrome was the second most common single major anomaly, in 14 infants (13%).

**Table 1:** Basic characteristics of participants and primary outcome.

Characteristics	Major anomalies	No major anomalies	*P*
	n = 109	n = 626	
Paternal			
Paternal age (yr)	32.9 ± 7.2	32.8 ± 6.6	0.90
Maternal			
Maternal age (yr)	30.8 ± 5.6	31.2 ± 5.5	0.42
Diabetes mellitus, n (%)	4 (3.7)	42 (6.7)	0.29
Chronic hypertension, n (%)	8 (7.3)	57 (9.1)	0.55
Antiepileptic drug intake*, n (%)	1 (0.9)	3 (0.5)	0.48
Cigarette smoking*, n (%)	1 (0.9)	19 (3.0)	0.34
Alcohol consumption*, n (%)	0 (0.0)	3 (0.5)	1.0
Primiparity, n (%)	49 (45)	339 (54)	0.076
Gestational age (wk)	38.5 ± 1.7	37.7 ± 1.8	< 0.001
Cesarean delivery, n (%)	27 (25)	184 (29)	0.33
Neonatal			
Male, n (%)	47 (43)	342 (55)	0.026
1-min Apgar score < 3, n (%)	3 (2.8)	14 (2.2)	0.73
5-min Apgar score < 7, n (%)	8 (7.3)	24 (3.8)	0.098
Umbilical artery pH	7.30 ± 0.08	7.29 ± 0.09	0.32
Birth weight (g)	2808 ± 484	2695 ± 552	0.029
Small for gestational age, n (%)	19 (17)	119 (19)	
Appropriate for gestational age, n (%)	81 (74)	427 (68)	
Large for gestational age, n (%)	9 (8.3)	80 (13)	
Placental weight (g)	554 ± 119	560 ± 129	0.66
Primary outcome			
Birth weight to placental weight ratio	5.18 ± 0.92	4.90 ± 0.78	0.003
Adjusted birth weight to placental weight ratio**	5.02 (4.87 − 5.17)	4.91 (4.85 − 4.97)	0.20

* During the first trimester of pregnancy

** Estimated mean (95% confidence interval) adjusted for gestational age and sex using ANCOVA

**Table 2:** Subgroups of major anomalies and associated anomalies.

EUROCAT subgroups	Major anomalies	Associated anomalies
	n = 109	n = 30
Nervous system (n = 5)	Myelomeningocele (2)	-
	Colpocephaly (1)	-
	Hydrocephalus (1)	-
	Spina bifida (1)	-
Eye (n = 0)	-	-
Ear, face and neck (n = 0)	-	-
Congenital heart defects (n = 37)	VSD (25)	Congenital hydronephrosis (2)
	Tetralogy of Fallot (3)	-
	Double outlet right ventricle (2)	Congenital hydronephrosis (1)
	PDA (2)	-
	TAPVR (2)	-
	ASD + VSD (1)	-
	Hypoplastic right heart (1)	-
	Pulmonary valve atresia (1)	-
Respiratory system (n = 1)	Pulmonary sequestration (1)	-
Orofacial clefts (n = 12)	Cleft lip with cleft palate (7)	-
	Cleft lip (3)	-
	Cleft palate (2)	VSD (1)
Digestive system (n = 10)	Intestinal malrotation (3)	-
	Small intestinal atresia (2)	-
	Anal atresia (1)	-
	Biliary atresia (1)	-
	Duodenal atresia (1)	-
	Esophageal atresia (1)	-
	Hirschsprung disease (1)	-
Abdominal wall defects (n = 0)	-	-
Urinary system (n = 6)	Congenital hydronephrosis (4)	-
	Renal hypoplasia (2)	-
Genital system (n = 0)	-	-
Limb (n = 3)	Club foot (1)	-
	Limb defect (1)	-
	Polydactyly (1)	-
Other anomalies/syndromes (n = 17)	Epidermolysis bullosa hereditaria (2)	-
	Spinal muscular atrophy (2)	-
	Beckwith-Wiedemann syndrome (1)	-
	Bloch-Sulzberger syndrome (1)	-
	Cornelia de Lange syndrome (1)	Hypospadias(1)
	Goldenhar syndrome (1)	ASD (1)
	Lowe syndrome (1)	-
	Myotubular myopathy (1)	-
	Noonan syndrome (1)	-
	Pena Shokeir syndrome (1)	-
	Pfeiffer syndrome (1)	-
	Pierre Robin syndrome (1)	-
	Situs inversus (1)	Single ventricle (1)
	Sotos syndrome (1)	ASD (1)
	Williams syndrome (1)	-
Chromosomal abnormality (n = 18)	Down syndrome (14)	PDA (6)
		ASD (4)
		Atrioventricular septal defect (3)
		VSD (2)
		Anal atresia (1)
		Polydactyly (1)
	Trisomy 18 (2)	Double outlet right ventricle (2)
		Esophageal atresia (1)
		Polydactyly (1)
	Trisomy 18 + XYY (1)	Double outlet right ventricle (1)
	Trisomy 9q + 10q26 deletion (1)	-

ASD, Atrial septal defect; PDA, Patent ductus arteriosus; TAPVR, Total anomalous pulmonary venous return; VSD, Ventricular septal defect

Following adjustment for gestational age and sex, the association between major anomalies and BW/PW ratio was analyzed. No difference in BW/PW ratio was seen between groups with or without major anomalies (Table 1) and the three categories of BW/PW ratios were equally distributed between the groups (Table 3). The prevalence of major anomalies was 17/141 (12%) in the <10th percentile of BW/PW ratio, 86/570 (15%) in the 10–90th percentile, and 6/24 (25%) in the >90th percentile. The number needed to diagnose a major anomaly varied between the three groups, with 8.3 in the <10th percentile of BW/PW ratio, 6.7 in the 10–90th percentile and 4.0 in the >90th percentile. The highest proportion of infants with major anomalies was observed in the >90th percentile of BW/PW ratio.

**Table 3:** Association between major anomalies and birth weight to placental weight ratio categories.

	Major anomalies	No major anomalies	*P*
	n = 109	n = 626	
<10th percentile, n (%)	17 (16)	124 (20)	0.30
10–90th percentile, n (%)	86 (79)	484 (77)	0.72
>90th percentile, n (%)	6 (5.5)	18 (2.9)	0.15

The distribution of major anomaly subgroups according to the three BW/PW ratio categories was analyzed (Table 4). Subgroups that included the nervous system, congenital heart defects, and orofacial clefts showed equally distributed trend across the three BW/PW ratio categories. On the other hand, subgroups of the digestive system, other anomalies/syndromes, and chromosomal abnormality showed predominant trend in the <10th percentile of BW/PW ratio.

**Table 4:** Distribution of major anomaly subgroups according to BW/PW ratio categories.

	Birth weight to placental weight ratio with major anomalies			*P*
	<10th percentile	10–90th percentile	>90th percentile	
	n = 17	n = 86	n = 6	
EUROCAT subgroups				
Nervous system, n (%)	1 (5.9)	3 (3.5)	1 (17)	0.32
Congenital heart defects, n (%)	2 (12)	32 (37)	3 (50)	0.089
Respiratory system, n (%)	1 (5.9)	-	-	0.065
Orofacial clefts, n (%)	1 (5.9)	9 (10)	2 (33)	0.17
Digestive system, n (%)	3 (18)	7 (8.1)	-	0.34
Urinary system, n (%)	1 (5.9)	5 (5.8)	-	0.83
Limb, n (%)	1 (5.9)	2 (2.3)	-	0.65
Other anomalies/syndromes, n (%)	2 (12)	15 (17)	-	0.47
Chromosomal abnormality, n (%)	5 (29)	13 (15)	-	0.19
Total	17 (100)	86 (100)	6 (100)	

## Discussion

This study is the first to report the BW/PW ratio in infants with major congenital anomalies and revealed a particular BW/PW ratio trend in each of the major anomaly subgroups. Compared with the general population, the group of infants in this study showed a tendency towards a low BW/PW ratio, and no difference was seen between singletons born with or without major anomalies. Comparing the three BW/PW categories, the proportion of infants with major anomalies was higher in the >90th percentile of BW/PW ratio. Among these BW/PW ratio categories, the major anomaly subgroup distribution showed that the nervous system, congenital heart defects and orofacial clefts exhibited evenly distributed trend across the three categories, while digestive system, other anomalies/syndromes and chromosomal abnormality exhibited predominantly distributed trend in the smallest BW/PW ratio category.

The association between the BW/PW ratio and perinatal outcomes has been actively investigated [10,11]. Among infants admitted to an NICU, the proportion of both a high BW/PW ratio (>90th percentile) and a low BW/PW ratio (<10th percentile) has been observed to be increased compared to a normal BW/PW ratio (10–90th percentile) [10]. A high BW/PW ratio (relatively small placenta) was associated with an increased risk of cerebral palsy in full-term births [11]. This suggests that a small placenta with a reduced surface area for the uptake of oxygen from the maternal circulation leads to insufficient oxygen supply to the fetal brain, resulting in cerebral palsy. In contrast, a low BW/PW ratio (relatively large placenta) was associated with cerebral palsy among preterm births [11]. A possible explanation is that the suboptimal condition of the fetus induced compensatory placental enlargement and a predisposition to preterm birth. Some congenital malformations including those with VACTERL association showed severe fetal growth restriction due to somatic hypocellularity [18]. In our study, a low BW/PW ratio was identified within the major anomaly subgroups of other anomalies/syndromes and chromosomal abnormality, which may be caused by fetal growth restriction. On the other hand, a mid-range or relatively high BW/PW ratio was observed within subgroups of congenital heart defects and orofacial clefts in the present study, which seems to be normal fetal growth explained by the lack of a profound associated anomaly.

Previous studies have demonstrated that fetal growth restriction was associated with chromosomal abnormality [19], VACTERL association [18], congenital heart defects [20], anencephaly [13], gastroschisis [13], esophageal atresia [13], and renal aplasia [13]. However, the association between congenital anomalies and the BW/PW ratio remains unknown. Only one previous study has investigated the relationship between congenital heart defects and the BW/PW ratio [21], where the BW/PW ratio in infants with congenital heart disease was distributed normally and no association was observed, similar to the results reported here.

Our findings demonstrate that the BW/PW ratio exhibited different distribution among the major anomaly subgroups. This is biologically plausible, as the effects of fetal growth differed in each of the major anomaly subgroups. In the <10th percentile of BW/PW ratio, the prevalence was comparatively higher among infants with abnormalities of the digestive system, other anomalies/syndromes, or chromosomal abnormalities. Severe fetal growth restriction was likely to occur in infants born with these profound congenital anomalies. In addition, because these fetal anomalies more often result in abortion or fetal death, a higher prevalence may be identified through ante-partum evaluation of growth-restricted fetuses. Estimated fetal weight and placental volume can be measured ultrasonographically during pregnancy [22]. Relatively enlarged placental volume accompanied by polyhydramnios and fetal morphological defects suggested fetal anomalies, such as anomalies of the digestive system, other anomalies/syndromes and chromosomal abnormality [23]. Conversely, relatively small placental volume and fetal malformation indicated fetal anomalies, such as congenital heart defects and orofacial clefts [15,24]. These abnormal ultrasonographic findings during pregnancy could predict the occurrence of congenital anomalies, facilitating the establishment of strategies for diagnosing and treating anomalies after birth.

The findings of this study must be considered in light of some limitations. First, PW in this study was measured without removing the umbilical cord. The only previous study to report reference values for PW and BW/PW ratio in Japanese measured PW without removal of the umbilical cord [9]. The same measurement procedure was therefore applied to this study to compare BW/PW ratio in Japanese. Umbilical cord weight was approximately 7% of PW [25], and BW/PW ratio differed by less than 5% among term pregnancies, regardless of the measurement procedure [4,7,9]. Second, this study did not include aborted or stillborn fetuses with major anomalies, because BW/PW ratio in these cases was thought to be skewed by surgical abortion or hemodynamic changes. In the study period, only seven fetuses with major anomalies were aborted in our center. Third, the sample size was limited, and subjects from only a single NICU were analyzed. However, all complicated pregnant women and neonates are transferred to our center in the region. Surveillance in our center thus reflects the regional epidemiological evidence. Multicenter studies with larger sample sizes may better elucidate the detailed association between subgroups of major anomalies and the BW/PW ratio.

In conclusion, the distribution of the BW/PW ratio differed among the subgroups of major anomalies. Infants with a suspected congenital disease were more likely to have major anomalies if BW/PW ratio was >90th percentile. Measurement of the BW/PW ratio is a valid assessment for diagnosing anomalies in infants.

## Supporting information

S1 Fig Diagnostic and classification process for major anomaly. (TIFF)

## Abbreviations

BW: Birth weight
BW/PW ratio: birth weight to placental weight ratio
NICU: neonatal intensive care unit
PW: placental weight
