# Supplementary figures and images for: Relationship between birth weight to placental weight ratio and major congenital anomalies in Japan
Source: PLoS One. 2018 Oct 22;13(10):e0206002. doi: 10.1371/journal.pone.0206002 (PMC6197685; doi:10.1371/journal.pone.0206002)

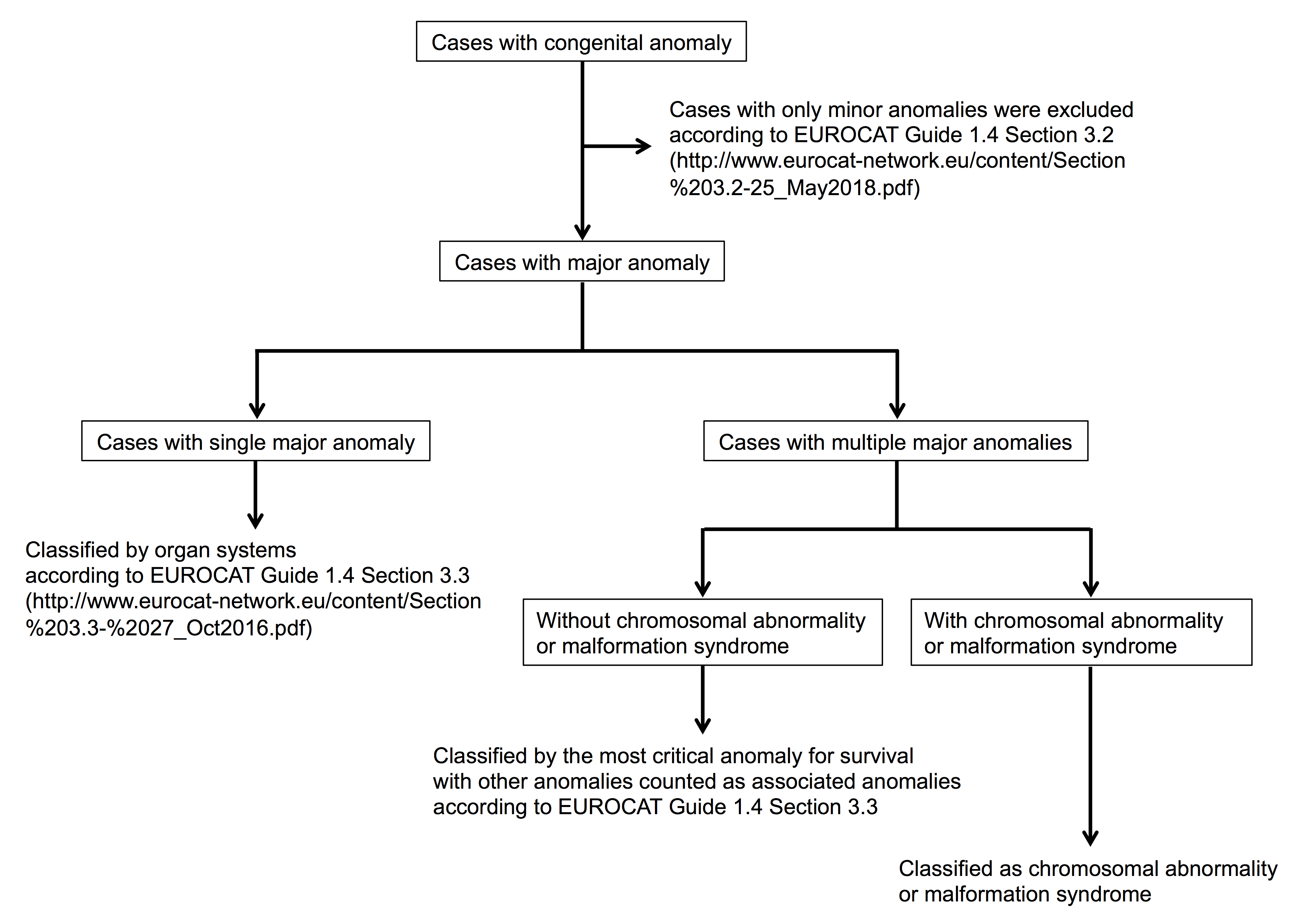

Supplement: S1 Fig — (TIFF) [file pone.0206002.s001.tiff]
